# Benign nasopharyngeal teratoma in an adult patient

**DOI:** 10.1016/S1808-8694(15)30588-7

**Published:** 2015-10-19

**Authors:** Lucas Gomes Patrocinio, Tomas Gomes Patrocinio, Sonia Regina Coelho, José Antonio Patrocinio

**Affiliations:** 1 Otorhinolaryngologist - Otorhinolaryngology Department - Federal University of Uberlândia Medical School.; 2 MD. ENT Resident - Otorhinolaryngology Department - Federal University of Uberlândia Medical School.; 3 MSc. Physician at the Otorhinolaryngology Department - Federal University of Uberlândia Medical School.; 4 Full Professor, Head of the Otorhinolaryngology Department - Federal University of Uberlândia Medical School. Otorhinolaryngology Department - Federal University of Uberlândia Medical School. Uberlândia, Minas Gerais, Brasil.

**Keywords:** adult, nasopharyngeal neoplasms, teratoma

## INTRODUCTION

The benign nasopharyngeal teratoma is a benign and rare congenital disease, made up of the three embryological leaflets (ecto, meso and endoderm). It is of slow and progressive growth, pressing against adjacent structures without invading them^1^. Its diagnosis is usually prenatal or during early childhood, due to ultrasound findings or severe obstructive respiratory symptoms. It is rare in adults, and there are only two of these cases in adults reported in the literature^2^, ^3^.

The goal of the present paper is to present the case of an adult patient with a nasopharyngeal teratoma, who was submitted to endoscopic surgery for exeresis.

## CASE REPORT

**Figure 1:**
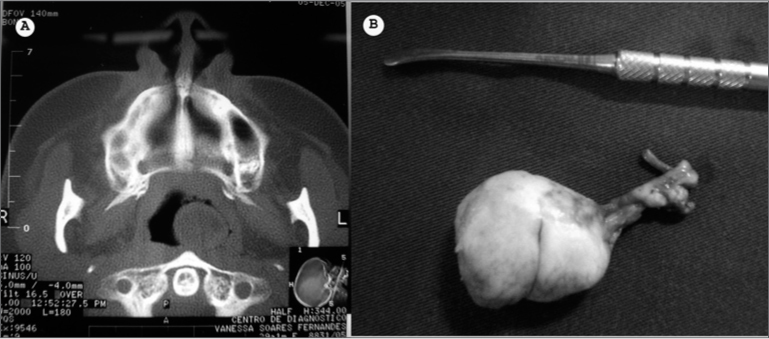
CT scan showing the nasopharynx teratoma (A) and the surgical specimen (B).

V.S., 35 years, female, presented unilateral nasal obstruction since childhood. Video-nasofibroscopy showed a pedicled oval-shaped mass in the pharyngeal ostium of the Eustachian Tube, obstruction about 60% of her nasopharynx. Paranasal sinuses CT scan presented a mass in her left nasopharynx (Figure 1a).

The patient was submitted to endoscopic surgery under local anesthesia, with lesion exeresis after pedicle identification and resection (Figure 1b). The tumor removed measured 4x3x2cm, similar to fat tissue at touch and with smooth surface. The pathology exam reported a benign mature teratoma of the nasopharynx.

The patient is being followed up in our outpatient ward for 7 months now, without recurrence of the lesion or respiratory symptoms.

## DISCUSSION

Teratomas are benign, cystic, semicystic or solid tumors, which derive from the three embryonic leaflets. Its prevalence in the population is of 1 to 4.000 newborns and only 2 to 5% of the cases are located on the head and neck. Most of these lesions are diagnosed at birth^4^.

Despite their controversial etiology, the most accepted theory is that these lesions stem from totipotent cells from the germinative layers of the embryo, and they grow in a potentially limited fashion and end up forming disorganized and disordered clusters^4^.

Symptoms depend on lesion size and location. The most frequent symptoms include nasal obstruction, dyspnea and difficulty to breastfeed, it may also cause difficulty to swallow and vomit. Intermittent symptoms such as cyanosis, nasal obstruction and dysphagia may also be present when the pedicle is long^1^.

Differential diagnosis of nasopharynx obstruction in the newborn include also choanal atresia, intranasal glioma and encephalocele, rhabdomyosarcoma, dermoid cyst, lymphangioma, hemangioma and other neurofibromatosis^5^, which must also be ruled out in adults.

CT scan and MRI help outline the lesion in order to plan for surgery, ruling out intracranial extension^3^, ^6^. Nasofibroscopy allows for early diagnosis in the assessment of nasal obstruction in newborns6. Treatment of choice for nasopharynx benign teratomas is surgical - complete lesion resection. Relapses are rare^4^.

In the case hereby reported, CT scan and the endoscopic exam were enough to assess lesion origin and extension. Clinical signs and symptoms of unilateral nasal obstruction should raise suspicion about neoplastic processes. Surgery was carried out with the 30° rigid scope, and the entire lesion was removed from the pharyngeal ostium of the Eustachian tube.

## CONCLUSION

Although rare, teratomas may affect the nasopharynx. We stress the importance of a careful exam of patients complaining of unilateral nasal obstruction, especially newborns. Surgical treatment can be carried out through the endoscopic approach and complete lesion exeresis according to preoperative planning guided by image exams.
